# Retrograde balloon pull-through technique for benign esophageal strictures: a single-center pilot experience

**DOI:** 10.1016/j.igie.2024.03.002

**Published:** 2024-03-26

**Authors:** Shae Patel, Andrew Wright, Paul Leonor, Wasseem Skef

**Affiliations:** 1 Department of Medicine, Loma Linda University Medical Center, Loma Linda, California, USA; 2 Section of Gastroenterology, VA Loma Linda Healthcare System, Loma Linda, California, USA; 3 Division of Gastroenterology and Hepatology, Loma Linda University Medical Center, Loma Linda, California, USA; 4 Division of Gastroenterology and Hepatology, Department of Medicine, Baylor College of Medicine, Houston, Texas, USA

## Abstract

**Background and Aims:**

Antegrade savary dilation and static balloon dilation are the mainstays of management of simple and complex benign esophageal strictures (BESs). A modified technique, termed retrograde balloon dilation, has potential advantages for the management of BESs. Efficacy and safety data on this technique are limited. We report a single-center experience of retrograde balloon dilation for BESs.

**Methods:**

We conducted a retrospective study evaluating retrograde balloon and antegrade savary dilation for BESs in 53 unique patients who met inclusion criteria, including 23 undergoing a retrograde balloon pull-through technique and 30 undergoing antegrade savary dilation. The primary endpoint was technical success, defined as achieving a luminal diameter of ≥16 mm. Secondary endpoints were repeat dilation rates within 1 year after achieving therapeutic endpoint dilation and adverse events.

**Results:**

Technical success was achieved in 22 of 23 patients (95.7%) with the retrograde balloon pull-through technique and in all 30 patients (100%) with antegrade savary dilation (*P* = .434). A nonsignificant trend of lower repeat dilation rates was present for the retrograde balloon pull-through group, with 4 of 22 in the retrograde balloon pull-through group versus 12 of 30 in the antegrade savary dilation group (*P* = .076). Only 1 minor adverse event occurred in the retrograde balloon pull-through group.

**Conclusions:**

Our experience suggests that retrograde balloon pull-through dilation is effective and safe for simple and complex benign esophageal stenosis.

Benign esophageal strictures (BESs) are nonmalignant stenoses of the esophageal lumen. Most esophageal strictures are secondary to GERD, although a variety of other conditions can also cause esophageal obstruction. BESs are typically categorized as simple (≤2 cm in length, straight, distal location) or complex (>2 cm in length, tortuous, severe narrowing, angulation).^1^ Endoscopic dilation is the mainstay of management, and BESs can typically be successfully and safely treated with either bougie or balloon dilation.

Although equally effective,^2^ the 2 dilation techniques have important distinctions. Bougie dilation works by exerting a longitudinal and radial force from the proximal to the distal end of the stenosis.^2^ In contrast, static balloon dilation depends solely on balloon inflation to generate a radial dilating force. Although theoretical advantages and disadvantages exist for both techniques, the selection of a particular dilator type is often based on stricture characteristics and the physician’s personal preference, training, and expertise.^3^ Static balloon dilation, however, can be limited in the setting of proximal or complex strictures.^4^ A variation of balloon dilation, retrograde balloon pull-through dilation,^5^^,^^6^ can potentially overcome these limitations. In this technique, the endoscopist inflates a through-the-scope dilation balloon distal to the stricture, anchors the balloon catheter to the endoscope with his or her finger, and then pulls the endoscope and balloon across the esophageal stricture. This allows dilation across the entire length of the stenosis and esophageal lumen, exerts both radial and longitudinal force, provides haptic feedback, and allows real-time estimation of the luminal diameter of the esophagus throughout its entire length with direct visualization of the dilation effect.

At our center, we adopted this technique for simple and complex BESs because of the abovementioned advantages. We hypothesized that this balloon pull-through technique would have similar efficacy and safety as savary dilation for the management of BESs.

## Methods

### Inclusion and exclusion criteria

We conducted a retrospective, single-center, cohort study of patients aged ≥18 years who underwent retrograde balloon pull-through dilation or antegrade savary dilation on upper endoscopy at Loma Linda University Medical Center from January 1, 2017 and November 30, 2022. Indications for EGD were dysphagia, odynophagia, and/or GERD. If a BES was identified, dilation was performed at the discretion and preferred technique of the endoscopist. If dysphagia was reported without overt stricture, empiric dilation was performed to rule out occult stricture. Patients with <1 year of follow-up, malignant stenosis, confirmed nonobstructive dysphagia (ie, achalasia), nonesophageal stenosis (ie, gastrojejunal stricture), or who underwent any alternate balloon dilation technique (ie, pneumatic, EsoFLIP [Medtronic, Minneapolis, Minn, USA] static balloon dilation) were excluded.

### Perioperative and operative care

Procedures were performed with patients under both moderate sedation and monitored anesthesia care. The decision to perform either sedation technique was at the discretion of the endoscopist and based on anesthesia availability, procedure complexity, and patient comorbidities. Periprocedural management of antithrombotic agents was per gastroenterology society guidelines.^7^ Postprocedure instructions regarding diet and medication resumption were relayed by the physician and nursing team, and patients were provided with written instructions. A postprocedure follow-up call by a dedicated registered nurse was completed for all patients within 48 hours as per established GI laboratory protocols. The goals of dilation were to relieve dysphagia and achieve a luminal diameter ≥16 mm in 1 or more sessions. If a dilation endpoint was not reached at the index endoscopy, a serial repeat endoscopy was scheduled for <4 weeks.

### Procedure technique

**Figure 1:**
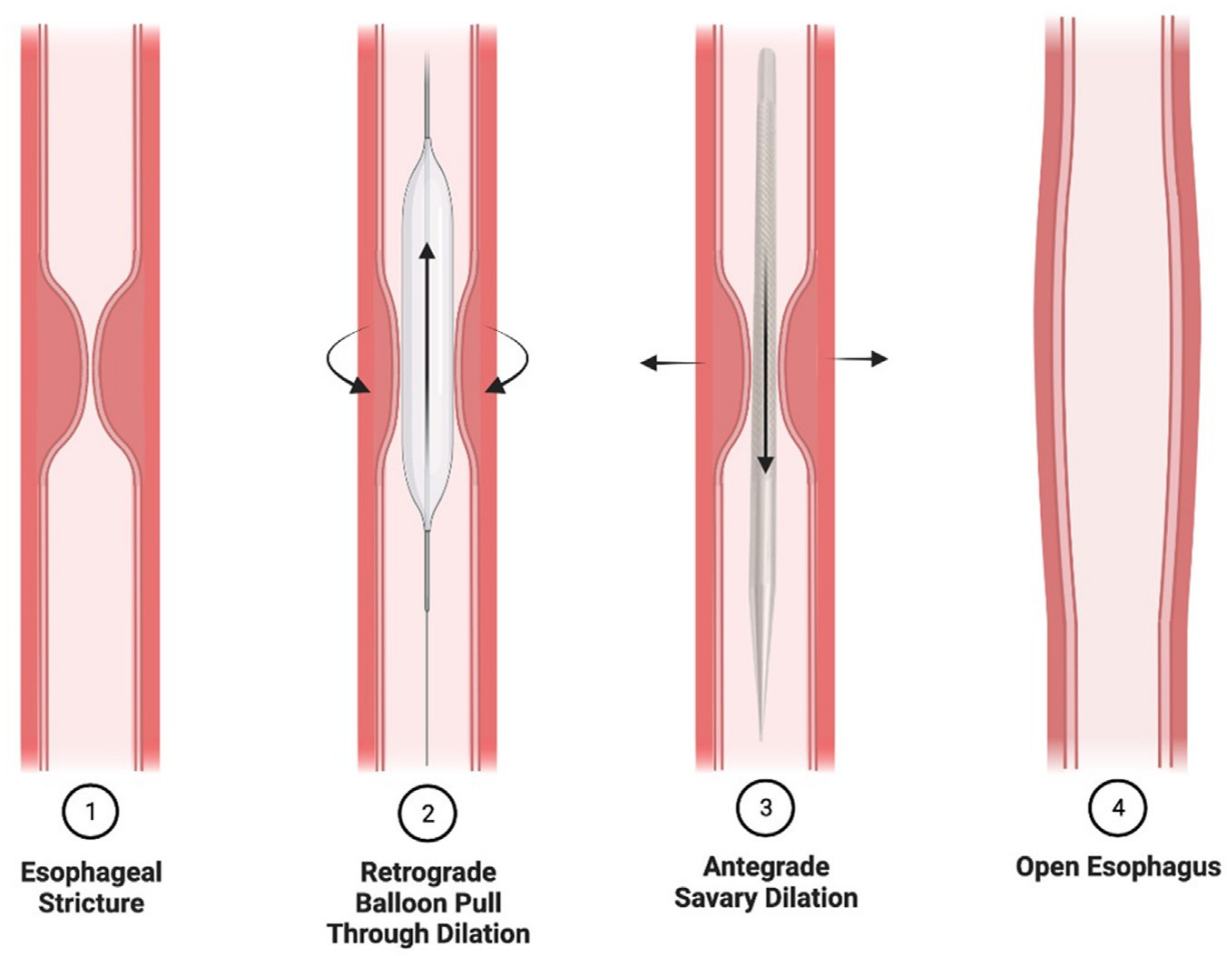
(**1**) Benign esophageal stenosis. (**2**) Retrograde dilation using a through-the-scope balloon dilator. (**3**) Antegrade savary dilation. (**4**) Post-dilation improvement in stricture diameter.

Antegrade bougie dilation was performed using a standard technique with reusable, flexible Savary-Gilliard (Wilson-Cook Inc, Winston-Salem, NC, USA) dilators over a guidewire with reinspection after dilation(s) to assess for outcome. Retrograde balloon pull-through dilation was performed by 3 endoscopists experienced with this technique using a CRE PRO Wireguided balloon dilatation catheter (Boston Scientific, Marlborough, Mass, USA) (Fig. 1). The technique involved assessing and traversing the stricture, if present, endoscopically.

After estimating the stricture diameter by comparing the lumen with the endoscope, the balloon was then inflated with water to the diameter of the stricture at the gastroesophageal junction, pulled backward to the tip of the endoscope, and both the endoscope and distended balloon were pulled proximally through the stenosis and along the length of the esophageal lumen under direct visualization. The pull-back speed was approximately 5 to 10 cm/s. The endoscopist’s left little finger clasped the catheter to maintain apposition of the balloon to the tip of the endoscope and to gauge resistance. If no overt stricture was identified but dysphagia was reported, empiric dilation of ≥18 mm was performed to rule out occult stricture. If severe stenosis was present, precluding the passage of a diagnostic endoscope (9.9 mm outer diameter), the stricture was traversed after downsizing to a GIF-XP190N endoscope (Olympus, Center Valley, Pa, USA). A guidewire was then passed into the stomach and was exchanged and backloaded onto a diagnostic endoscope with a preloaded balloon dilation catheter tip projecting at the tip of the endoscope working channel. The diagnostic scope with dilation balloon was then inserted over the guidewire and advanced toward the stricture. The balloon was passed distal to the stricture and inflated to the desired diameter, and retrograde dilation was then performed by pulling back on the balloon under endoscopic visualization with direct haptic feedback. Serial balloon dilation was performed per session until the dilation endpoint was reached, as determined by the endoscopist (Fig. 1).

### Data collection and definitions

Data were collected from electronic medical records (Epic Systems Corporation, Verona, Wisc, USA) and endoscopy software (EndoSoft LLC, Schenectady, NY, USA). Baseline variables including age, sex, body mass index, ethnicity, history of perforation, sedation type, stricture type (simple or complex), etiology and location(s), presence of esophagitis (classified by Los Angeles grade), Barrett’s esophagus, use of antithrombotic therapy, dilation type and number of dilations performed per session, use of fluoroscopy, and number of dilation sessions were collected.

Our primary outcomes were relief of dysphagia and the ability to reach the therapeutic dilation target in 1 or more sessions. We defined the therapeutic endpoint dilation target as ≥16 mm,^8^ based on literature review. Secondary endpoints were redilation within 1 year of achieving therapeutic dilation target, time to repeat dilation (days), and adverse events according to established classification.^9^

### Statistical analysis

Baseline patient characteristics were compared by type of dilation (retrograde balloon pull-through dilation vs antegrade bougie dilation) using the χ^2^ test and Fisher exact test to compare categorical variables and the Wilcoxon rank sum test for continuous variables, respectively. A *P* < .05 was considered statistically significant. All statistical analyses were performed using SAS analytics software, version 9.4M8 (SAS Institute, Cary, NC, USA).

### Ethical statement

This study was approved by the institutional review board at Loma Linda University Medical Center (IRB no. 5220455). Written informed consent was waived for participating in this study.

## Results

### Cohorts and baseline variables

Of 53 patients who met inclusion criteria, 23 patients underwent the retrograde balloon pull-through technique and 30 patients underwent antegrade bougie dilation using a Savary dilator. Ten patients were lost to follow-up because of noncompliance, moving, or death. No differences were found in sex, history of perforation, sedation type, presence of esophagitis or Barrett’s metaplasia, stricture type, location or etiology of the stricture, baseline diameter, and antithrombotic therapy (Table 1). A nonsignificant trend of more white patients was found in the savary dilation group. No patients had a history of perforation or required fluoroscopy for dilations.

**Table 1:** Baseline patient characteristics

Characteristics	Balloon pull-through dilation (n = 23)		Savary dilation (n = 30)		*P* value
	No. of cases	Percentage	No. of cases	Percentage	
Sex					.586
Female	11	47.83	17	56.67	
Male	12	52.17	13	43.33	
Race/ethnicity					.063
Black or African American	2	8.70	1	3.33	
Hispanic	10	43.48	6	20.00	
White	11	47.83	23	76.67	
Indication for procedure					.839
Dysphagia	17	73.91	20	66.67	
Odynophagia	1	4.35	2	6.67	
GERD	5	21.74	8	26.67	
Barrett's esophagus					.218
Yes	5	21.74	2	6.67	
No	18	78.26	28	93.33	
Type of esophagitis					.778
Los Angeles grade A esophagitis	3	13.04	2	6.67	
Los Angeles grade B esophagitis	1	4.35	1	3.33	
Los Angeles grade C esophagitis	1	4.35	1	3.33	
Los Angeles grade D esophagitis	1	4.35	0	.00	
Location of stricture					.290
Upper esophagus only	2	8.70	4	13.33	
Middle esophagus only	3	13.0	2	6.67	
Lower esophagus only	13	56.52	22	73.33	
Two segments involved (middle and lower esophagus)	5	21.74	2	6.67	
Three segments involved	0	.00	0	.00	
Type of stricture					.489
Esophageal (ie, Schatzki’s) ring	8	34.78	13	43.33	
Anastomotic stricture	1	4.35	2	6.67	
Simple peptic stricture	8	34.78	12	40.00	
Complex peptic stricture	5	21.74	2	6.67	
Eosinophilic esophagitis	1	4.35	0	.00	
Caustic stricture	0	.00	1	3.33	
Sedation type					.261
Moderate	5	21.74	9	30.00	
Monitored anesthesia care	16	69.57	21	70.00	
General anesthesia	2	8.70	0	.00	
Use of anticoagulation					.642
Yes	2	8.70	2	6.67	
No	21	91.30	28	93.33	
Use of dual-antiplatelet therapy					.434
Yes	3	13.00	2	6.67	
No	20	86.96	28	93.33	
	Median	Interquartile range	Median	Interquartile range	
Initial diameter of benign esophageal stricture, mm	15	14-16	15.7	14.25-17	.328
Time of procedure, min	16.65	15-18	16.87	15-18	.830

### Primary and secondary endpoints

The therapeutic dilation target, defined as achieving a luminal diameter of ≥16 mm, was equal in both groups (Table 2). Twenty-two of 23 patients (95.7%) in the retrograde balloon pull-through dilation group and all 30 patients (100%) in the antegrade bougie dilation group achieved the primary endpoint (*P* = .434). No differences occurred in the number of endoscopic sessions required to achieve the endpoint (1.3 vs 1.57, *P* = .266) and in the procedure time (16.7 vs 16.9 minutes, *P* = .83) between both groups.

**Table 2:** Primary and secondary endpoints

Endpoint	Balloon pull-through dilation (n = 23)		Savary dilation (n = 30)		*P* value
	No. of cases	Percentage	No. of cases	Percentage	
Primary					
Therapeutic dilation achieved (dilation ≥16 mm)					.434
Yes	22	95.65	30	100	
No	1	4.35	0	.00	
Secondary					
Type of adverse events (per ASGE lexicon)					1.00
Mild	1	4.35	0	.00	
Moderate	0	0.00	0	.00	
Severe	0	0.00	0	.00	
Was redilation needed (1 year)?					.076
Yes	4	17.39	12	40.00	
No	19	82.61	18	60.00	
Total no. of endoscopies	30		48		
Compliance with rule of 3s	28	93.33	47	97.92	
	Median	Interquartile range	Median	Interquartile range	
No. of dilators used in 1 session	2	1-3	1	1-2	.210
No. of days to redilation	92	51-147	115	68-221	.515
	Mean	Interquartile range	Mean	Interquartile range	.266
No. of sessions to achieve the therapeutic endpoint	1.30	1-1.5	1.57	1-2	

Among those who achieved the therapeutic dilation endpoint, 4 patients (17.4%) required redilation in the retrograde balloon dilation cohort versus 12 (40%) in the antegrade bougie dilation cohort within the first year (*P* = .076) (Table 2). The median time to redilation was 92 days (interquartile range, 51-147) in the retrograde balloon pull-through group and 115 days (interquartile range, 68-221) in the antegrade bougie dilation group (*P* = .515). Only 1 adverse event occurred in the retrograde balloon pull-through dilation group: a deep mucosal tear that did not require any intervention (Table 2).

## Discussion

Here we report a single-center preliminary experience with a retrograde balloon dilation pull-through technique for BESs. Our study suggests that this technique is effective and safe for both simple and complex esophageal strictures.

A luminal diameter >13 mm has been suggested to be sufficient to relieve dysphagia symptoms in individuals with esophageal stenosis.^10^ Although the endpoint for BES dilation remains controversial,^11^ the minimum goal should be to relieve dysphagia and prevent stricture recurrence. Therefore, we chose an endpoint goal of 16 mm because data support an improved dilation-free period at this target diameter.^8^ Our study found that retrograde balloon pull-through dilation was effective at achieving the endpoint dilation and comparable with dilation with bougienage. Overall, our success rate for retrograde balloon pull-through dilation was consistent with published success rates of 83% to 100% for static balloon dilation and 78% to 100% for bougie dilation.^12^ In addition, our rates of redilation within 1 year in 4 of 19 patients (17.3%) were comparable with the existing literature.^8^ Finally, we observed only 1 minor adverse event, which is consistent with published safety data.^13^

The theoretical advantages of retrograde balloon pull-through over antegrade savary dilation merit consideration. Madanick et al^6^ proposed that this method allows a better estimation of the stenosis caliber and consequently aids in the selection of an appropriately sized dilation catheter, thereby limiting dilator use per session. Although theoretically advantageous, our series did not show a difference in the number of dilators used per session between either dilation techniques. However, we suggest additional advantages include dilation under direct visualization, increased efficiency, and patient tolerance with this technique. For example, it is known that bougie dilation is associated with increased patient discomfort compared with through-the-scope dilation,^14^ largely because the act of antegrade dilation is nonendoscopic and involves sequentially increasing diameter dilators with associated discomfort. Additionally, bougie dilation requires endoscope reintubations after the passage of each dilator, which is undesirable. One may argue that a theoretical disadvantage of retrograde balloon pull-through dilation is that the dilating force is not strictly radial but also targets the shelf of the stricture or wall of the esophagus with shear force without a tapered dilator, which is hypothesized to increase the risk of adverse events.^15^ However, our study did not demonstrate any difference in adverse events between both dilation techniques.

We further propose this technique may also be preferred for presumed nonobstructive dysphagia to rule out occult strictures. It is established that subtle BESs can be notoriously difficult to detect during upper endoscopy,^16^ especially in disorders such as eosinophilic esophagitis. Hence, empiric dilation can sometimes detect occult strictures in what would otherwise be mischaracterized as nonobstructive dysphagia.^17^ This is why empiric bougie dilation is sometimes performed and endorsed by esophagologists in the setting of dysphagia.^18^ In this setting, push-type bougie dilation is favored because the dilation effect is uniform along the entire length of the esophagus. Nevertheless, we suggest a retrograde technique is equally effective in presumed nonobstructive dysphagia to rule out occult BESs.

The strength of our study is that this is the largest series to examine retrograde dilation of which we are aware, and we report its use in different types of BESs. In addition, we used defined endpoints supported by the literature. Limitations of our study include its small sample size, single-center setting, and retrospective design with known shortcomings of confounding and loss of follow-up. We accept that the baseline stricture diameters were mild to moderate, and alternative techniques with established safety and efficacy may be preferred for severe strictures before adopting this technique. We also noted a nonsignificant difference in race between both groups, but this was coincidental and likely because of the small sample size. Moreover, we did not capture validated dysphagia questionnaires on patients, which would have been a compelling endpoint to compare. Additionally, we did not capture proton pump inhibitor therapy use, which is known to reduce the need for redilation for BESs.^19^

In conclusion, we report a single-center experience demonstrating the efficacy and safety of retrograde balloon pull-through dilation for mild to moderate BESs. Further prospective research with a larger sample sizes is essential to validate our findings and inform clinical practice.

## Disclosure

The following author disclosed financial relationships: W. Skef: Stock in GE Healthcare and Pfizer. All other authors disclosed no financial relationships.
